# Chronic rhinosinusitis is not associated with increased incidence of acute myocardial infarction: A national population-based study

**DOI:** 10.1371/journal.pone.0286048

**Published:** 2023-09-27

**Authors:** Hyun Jung Kim, Hyeong Sik Ahn, Ji-Hun Mo, Sumin Son, Seung Ho Kim, Ikhee Kim, Ki-Il Lee

**Affiliations:** 1 Department of Preventive Medicine, Korea University College of Medicine, Seoul, Republic of Korea; 2 Department of Otorhinolaryngology-Head and Neck Surgery, Dankook University College of Medicine, Cheonan, Republic of Korea; 3 Department of Otorhinolaryngology-Head and Neck Surgery, Konyang University College of Medicine, Daejeon, Republic of Korea; 4 Myunggok Medical Research Institute, Konyang University College of Medicine, Daejeon, Republic of Korea; Kyung Hee University School of Medicine, REPUBLIC OF KOREA

## Abstract

**Background & aims:**

Chronic rhinosinusitis (CRS) is one of the most prevalent upper respiratory tract diseases. However, little is known the effect of CRS on the cardiovascular aspects of patients. This study aimed to investigate the incidence of acute myocardial infarction (AMI) in patients with CRS compared with that in the general population.

**Methods:**

This retrospective cohort study was performed using the Korean National Health Insurance Service (NHIS) database. To minimize confounding, age, sex, and cardiovascular risk profiles were adjusted. The primary endpoint was newly diagnosed AMI in patients between January 2005 and December 2018. The relative risk of AMI in patients with CRS was compared with that in controls. Kaplan–Meier survival curves and Cox proportional regression tests were used for statistical analyses.

**Results:**

Among 5,179,981 patients from the NHIS database, 996,679 patients with CRS were selected. The control group was 10 times (n = 9,966,790) the number of individuals in the CRS group. The CRS group had better cardiovascular profiles than those of the control group and had an adjusted hazard ratio of 0.99 (95% confidence interval, 0.97–1.02) for AMI.

**Conclusion:**

There was no significant association between the two groups regardless of the presence of nasal polyps. This is the first study adjusting cardiovascular risk profiles and analyzing the relationship between CRS and AMI. CRS was not associated with a high incidence of AMI after adjusting for cardiovascular risk factors.

## Introduction

Chronic rhinosinusitis (CRS) is one of the most prevalent upper respiratory tract diseases and is characterized by local inflammation in the upper airway [1, 2]. Clinically, CRS is defined as the presence of over 12 weeks of symptomatic manifestations including nasal obstruction, postnasal drip, rhinorrhea, and facial pain associated with infected sinuses [3]. The etiology of CRS is considered to be primarily idiopathic [2]. CRS is known as a multi-factorial inflammatory disorder triggered by an anatomical blockage of the sinus ostium, allergy, viral and bacterial infections, or congenital abnormalities, such as immunodeficiency or primary ciliary dyskinesia. CRS with nasal polyposis results in reduced quality of life for patients and high costs for disease treatment [4]. In addition, CRS with nasal polyps is associated with an intrinsic Th2 inflammation of the airways [5].

CRS can be associated with systemic diseases [6], such as allergic [7], respiratory [8], gastrointestinal [9], and even neurodegenerative diseases [10]. However, these systemic associations are not fully elucidated as previous studies used a relatively small sample size or were evidence-based reviews.

Several observational cohort studies have reported a relationship between CRS and cardiovascular diseases. Hao et al. [11] and Wang et al. [12] reported that the risk of acute myocardial infarction (AMI) was increased in patients with CRS in a Taiwanese nationwide cohort study. In a population-based study in Korea [13], the estimated risk of AMI marginally increased in patients with CRS. Lee et al. [6] demonstrated that CRS increased the risk of cerebrovascular diseases, such as ischemic and hemorrhagic stroke, in another nationwide population-based case-control study in Korea.

However, the relationship between CRS and AMI remains questionable due to the reasons that follow. First, the risk of developing AMI in patients with CRS needs to be identified from the perspective of cardiovascular and metabolic risk profiles, as the two are closely associated. The aforementioned studies had limited data regarding cardiovascular and metabolic risk factors, and key risk factors, such as hypertension, diabetes, dyslipidemia, and smoking, were not controlled. Only a few studies have reported risk profiles among patients with CRS [12], but they also had restrictions due to the insufficient definition of cardiovascular risk being dependent on diagnostic codes. Second, relatively small sample sizes from representative national sample cohorts have been used. Third, the study participants were selected using prevalent cases rather than incident cases. Hence, the relationship between CRS and the incidence of AMI needs to be clarified through studies with large populations after controlling for risk profiles.

The Korean National Health Insurance Service (NHIS) includes a complete set of health data on approximately 50 million South Koreans and information on demographics, comorbidities, diagnostic codes with the International Classification of Disease-10 (ICD-10), and medical examination codes of procedures and prescriptions.

This study had two aims. First, we sought to compare the cardiovascular risk profiles, including metabolic profiles, among patients with CRS and matched controls. Second, we sought to determine whether CRS was associated with an increased risk of AMI in patients after adjusting for cardiovascular risk profiles.

## Materials and methods

### Study setting and population

This retrospective cohort analysis was based on the Korean NHIS claims database, including the National Health Screening Program (NHSP) data. The NHIS database includes information on patients’ baseline characteristics, such as age, sex, residence, socioeconomic status, comorbidities, ICD-10 diagnostic codes, and medical examination codes for procedures and prescriptions. The NHSP data contain health check-up information for the general population via the NHIS. Most adults are eligible for standardized medical examinations every 2 years. Approximately 80% of the cohort in this study underwent a national health check-up in Korea. For the mortality analysis, death registrations from the Korean National Statistical Office were merged into the NHIS database.

We collected the data of patients who were diagnosed with CRS between January 2005 and December 2018 from the NHIS database. Patients with CRS were defined as those who visited the out-patient clinic more than five times with an ICD code of J032 (Table 1). We analyzed all types of health facility diagnosing CRS and AMI (primary clinic, general hospital, and tertiary hospital). To improve the accuracy of the diagnostic code, only patients with CRS who were diagnosed by endoscopy (E7530, E7540, E7550, or E7560) were selected. The subgroup analysis was performed according to the presence of nasal polyps (NP).

**Table 1:** Working definition derived from the NHIS claims database.

Disease	Working definition
CRS	At least one claim under ICD-10 codes J32 + ≥ five out-patient visits within a year + nasal endoscopy (E7530, E7540, E7550 or E7560)
CRSsNP	At least one claim under ICD-10 code J32 + ≥ five out-patient visits without J33 + nasal endoscopy (E7530, E7540, E7550 or E7560)
CRSwNP	At least one claim under ICD-10 code J32 + ≥ five out-patient visits with J33 + nasal endoscopy (E7530, E7540, E7550 or E7560)
AMI	At least one claim under ICD-10 code I21 + ≥ five out-patient visits or ≥ one admission

Abbreviation: NHIS, Korean National Health Insurance Service; ICD-10, International Classification of Diseases, 10^th^ edition; CRSsNP, chronic rhinosinusitis without nasal polyps; CRSwNP, chronic rhinosinusitis with nasal polyps, AMI, acute myocardial infarction.

To investigate the incidence of AMI, a 3-year washout period was applied. The exclusion criteria were as follows: (a) patients who did not participate in a health check-up 3 years before the initial diagnosis, (b) patients who had been diagnosed with AMI, (c) patients who were younger than 20 years of age or older than 80 years of age, and (d) patients who died between 2005 and 2018 due to reasons other than AMI and CRS.

The control group comprised all individuals who participated in the NHSP between 2005 and 2018 and had not been officially diagnosed with CRS during the same period. The same exclusion criteria as those used for the CRS group were applied to the control group. We randomly selected controls (four per patient with CRS) after matching participant year-by-year with patients in the CRS group for age, sex, and the year of health check-up.

### Assessment of cardiovascular risk profiles

The cardiovascular risk profiles of the patients were acquired from the NHSP data. The variables included systemic factors, such as systolic and diastolic blood pressure (BP), fasting blood sugar (FBS) levels, body mass index (BMI), blood cholesterol levels, proteinuria, and environmental factors, such as physical activity, smoking status, and alcohol consumption. We analyzed the incidence rates of AMI in the CRS and control groups according to the sub-classified risk profiles. The cardiovascular risk profiles were acquired in the year of the CRS diagnosis or, if unavailable, within 2 years prior to the diagnosis. Each variable of the cardiovascular risk profile was categorized in both the groups.

### Outcome measurement

Newly diagnosed AMI in patients across 13 years, from January 1, 2005 to December 31, 2018, was the main outcome of interest in this study. AMI was defined as one in-patient admission with the ICD-10 code of I21 (AMI) or ≥ five out-patient visits [14]. AMI is included in the Special Support for Serious Illness (SSSI) act, which was launched by the Korean government and reduces the statutory coinsurance rate for registered patients. As registration requires a physician’s confirmation and an additional review by another healthcare professional to ensure that the illness meets the diagnostic criteria, the data regarding SSSI, including AMI, are considered reliable. The endpoint of this study was defined as death, a diagnosis of AMI, or the end of the study (December 31, 2018).

### Ethical approval

This study was approved (2020-05-022) by The Institutional Review Board (IRB) of Konyang University Hospital. The need for informed written consent was waived by the IRB because of the retrospective study design.

### Verification of the diagnosis

We analyzed data among 300 patients (100 patients with CRS, and 200 suspected non-CRS patients: 100 patients with septal deviation and 100 patients with chronic rhinitis) for the verification of the diagnosis. To identify patients with CRS using the diagnostic code, we performed the diagnostic verification according to the number of visits to the clinic (S1 Table). In order to assess the diagnostic accuracy, two otorhinolaryngologists reviewed the clinical information of the CRS and suspected non-CRS subjects. Additionally, the diagnostic accuracy including sensitivity, specificity, positive predictive value, and negative predictive value were determined. In a validation study, we found a sensitivity of 78% in 2 hospital visits and also a specificity of 100% in ≥5 hospital visits. A statistical expert decided the best specificity level of ≥5 hospital visits as a working definition.

In addition, our data were collected from whole nationwide information including all types of health facility. Therefore, there was minimal risk of clustering of data.

### Statistical analysis

We compared the cardiovascular risk profiles of patients with CRS and matched controls using standardized differences. We calculated the standardized mean differences between the groups using Stata software (version 12.0; Stata Corporation, College Station, TX, USA). An absolute standardized difference of 0.10 or more indicated that the covariates were imbalanced between the groups. The variables are categorized and presented as frequencies and percentages. Descriptive statistics and standard deviations were calculated to compare the categorical variables of the CRS and control groups [15].

The person-years for each individual in both the CRS and control groups were calculated from the start to the end dates of follow-up. The hazard ratio (HR) of AMI, stratified by age and sex, was calculated as the number of AMI per 1000 person-years. The Kaplan–Meier plot was used to analyze the cumulative incidence of AMI events between the control and CRS groups [16].

A multivariable stratified Cox proportional hazards regression model, considering a matched study design, was used to examine the association between CRS and the risk of AMI events [17]. The HRs adjusted for cardiovascular risk profiles (systolic BP, diastolic BP, FBS, cholesterol level, proteinuria, BMI, alcohol consumption pattern, and smoking status) and the corresponding 95% confidence intervals (CIs) for the estimation of the associations between CRS and AMI are shown. The severity-dependent associations between cardiovascular risk factors and the HR of AMI were analyzed using a forest plot. Moreover, the forest plot was used to analyze the adjusted HR (95% CI) of AMI according to age, sex, and presence of NP.

All statistical analyses were performed using the Stata software. Statistical significance was set at a two-tailed 95% CI, and *P*-values < 0.05 were considered statistically significant. Propensity score matching was conducted using Stata software with a significance level of 0.05 [18]. Missing data were handled with multiple imputation using the chained equation method [19, 20].

## Results

### Baseline characteristics of subjects

Between January 1, 2005 and December 31, 2018, 5,179,981 patients were newly diagnosed with CRS. Of these, 996,679 patients with CRS were evaluated. For the control group, 9,966,790 participants were recruited from the general population. Overall, 47.84% of the patients were female, and 49.34% were >50 years of age at the time of diagnosis. There was a lower proportion of patients with high BP and pulse rate in the CRS group than in the control group (BP, 15.26% and 16.46%; pulse rate, 10.60% and 11.52%, respectively). Blood total cholesterol, FBS levels, and proteinuria levels were also lower in the CRS group than in the control group (blood cholesterol, 10.97% and 11.36%; FBS, 6.22% and 6.86%; proteinuria, 2.14% and 2.23%, respectively). Increased alcohol consumption was lower in the CRS group than in the control group (12.59% and 13.52%, respectively). Patients with CRS had a higher frequency of increased physical activity than that of patients in the control group (20.03% and 19.03%, respectively). The proportion of current smokers was similar in the CRS and control groups (44.76% and 44.74%, respectively). Detailed data regarding the baseline characteristics are presented in Table 2.

**Table 2:** Cardiovascular risk profiles in patients with CRS and control group.

Variables	Controls (n = 9,966,790)	Patients with CRS (n = 996,679)	Standardized mean difference
Sex			0.00
Male	5,198,590 (52.16)	519,859 (52.16)	
Female	4,768,200 (47.84)	476,820 (47.84)	
Age (years)			0.00
20–29	884,970 (8.88)	88,497 (8.88)	
30–39	1,876,900 (18.83)	187,690 (18.83)	
40–49	2286850 (22.94)	228,685 (22.94)	
50–59	2,457,650 (24.66)	245,765 (24.66)	
60–69	1,720,880 (17.27)	172,088 (17.27)	
70–79	739,540 (7.42)	73,954 (7.42)	
Blood pressure (mmHg)			0.04
SBP <120, DBP <80	3,651,320 (36.63)	374,937 (37.62)	
SBP ≥120 and <130, DBP <80	1,161,825 (11.66)	119,218 (11.96)	
SBP ≥130 and <140, DBP ≥80 and <90	3,510,121 (35.22)	350,112 (35.13)	
SBP ≥140 and <180, DBP ≥90 and <120	1,595,465 (16.01)	148,941 (14.94)	
SBP ≥180, DBP ≥120	45,293 (0.45)	3,181 (0.32)	
FBS (mg/dL)			0.03
<110	8,389,461 (84.17)	847,058 (84.99)	
≥110 and <126	889,376 (8.92)	87,201 (8.75)	
≥126	683,434 (6.86)	61,962 (6.22)	
Total cholesterol (mg/dL)			0.02
<200	5,676,565 (56.95)	574,713 (57.66)	
200-<240	2,976,403 (29.86)	294,430 (29.54)	
≥240	1,132,028 (11.36)	109,341 (10.97)	
Proteinuria (grading)			0.01
<3+	9,701,085 (97.33)	971,664 (97.49)	
≥3+	221,978 (2.23)	21,323 (2.14)	
Physical activity (per week)			0.03
None	4,766,489 (47.82)	463,828 (46.54)	
<3	2,925,547 (29.35)	295,552 (29.65)	
≥3	199,587 (19.03)	1,896,274 (20.03)	
Smoking (pack years)			0.02
none	5,323,082 (53.41)	532,697 (53.45)	
<5	921,816 (9.25)	90,552 (9.09)	
≥5 and <10	864,482 (8.67)	85,037 (8.53)	
≥10 and <20	1,276,657 (12.81)	125,617 (12.60)	
≥20 and <30	718,107 (7.20)	72,982 (7.32)	
≥30	678,160 (6.80)	71,936 (7.22)	
Alcohol consumption (per week ^a^)			0.03
None	3,976,402 (39.90)	402,993 (40.43)	
< 1	2,135,563 (21.43)	211,380 (21.21)	
≥1 and < 4 (female), ≥1 and < 5 (male)	549,680 (5.52)	53,663 (5.38)	
≥4 (female) and ≥5 (male)	1,347,987 (13.52)	125,438 (12.59)	
BMI (kg/m^2^)			0.03
<18.5	370,760 (3.72)	33,876 (3.40)	
≥18.5 and <23.0	3,810,560 (38.23)	368,536 (36.98)	
≥23.0 and <25.0	2,425,991 (24.34)	249,148 (25.00)	
≥25.0			

Abbreviations: CRS, chronic rhinosinusitis; SBP, systolic blood pressure; DBP, diastolic blood pressure; FBS, fasting blood sugar; BMI, body mass index.

^a^ 250 mL = a cup of beer

### Risk factors for AMI events by cox proportional hazard model

**Fig 1:**
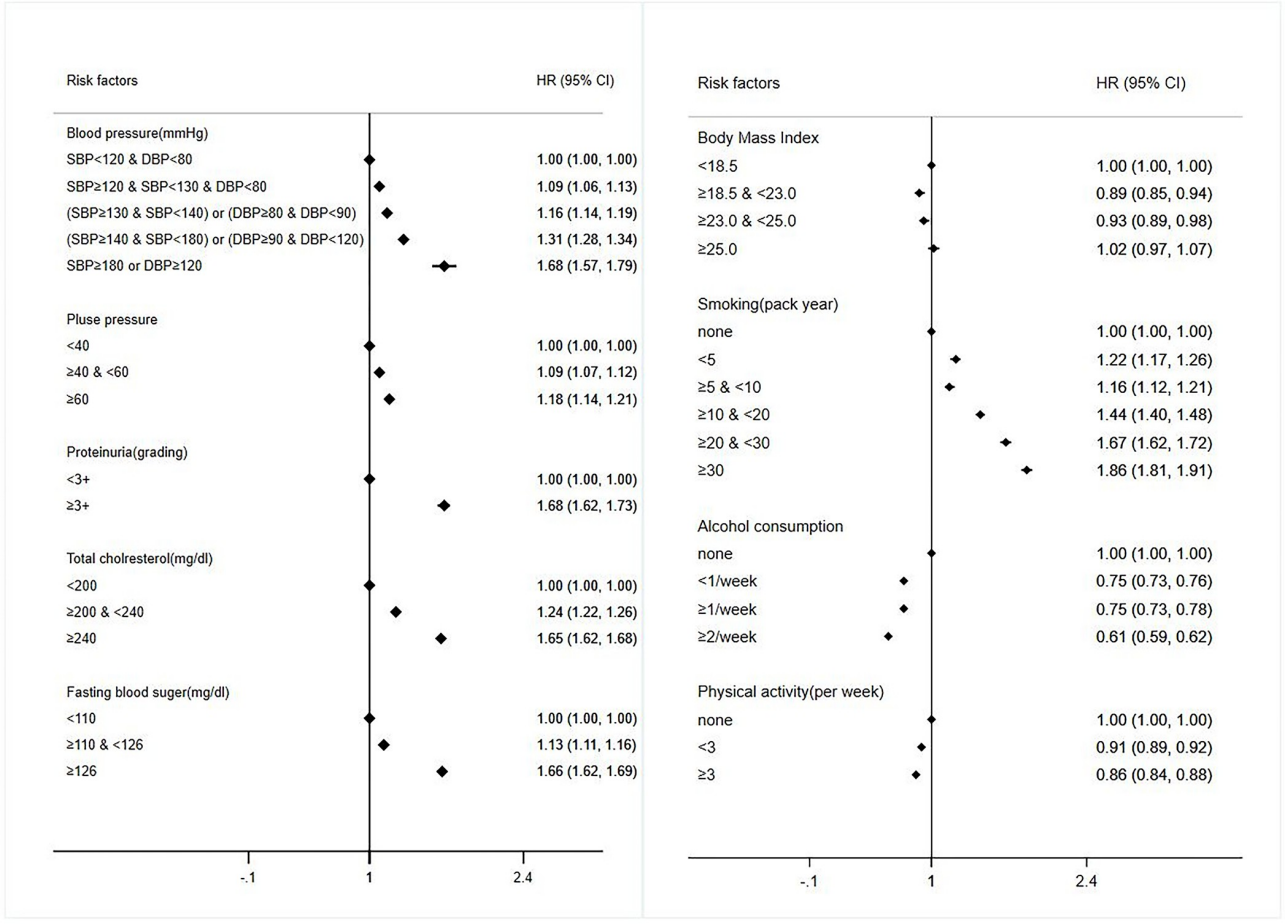
Adjusted hazard ratios in patients with CRS for AMI according to cardiovascular risk factors. Risk factors such as hypertension, diabetes, dyslipidemia and smoking history were independently associated with AMI. On the other hand, the HR values of AMI were attenuated in those with increased physical activity compared with those without physical activity. HR, hazard ratio; CI, confidence interval; SBP, systolic blood pressure; DBP, diastolic blood pressure.

According to several covariates, the adjusted HRs of AMI were: 1.68 (1.57–1.79) in ≥180 mmHg systolic BP and ≥120 mmHg diastolic BP; 1.66 (1.62–1.69) in ≥ 126 mg/dL FBS; 1.68 (1.62–1.73) in proteinuria; 1.65 (1.62–1.68) in ≥ 240 mg/dL blood cholesterol; and 1.86 (1.81–1.91) in ≥ 30 pack-years of smoking history. Hypertension, diabetes, dyslipidemia, and smoking history were independently associated with AMI. The HR values of AMI were attenuated in those with increased physical activity (0.86 [0.84–0.88]) compared with those with no physical activity under the same conditions. Detailed results are shown in Fig 1.

### Comparison of the incidence of AMI between the CRS and control groups

**Fig 2:**
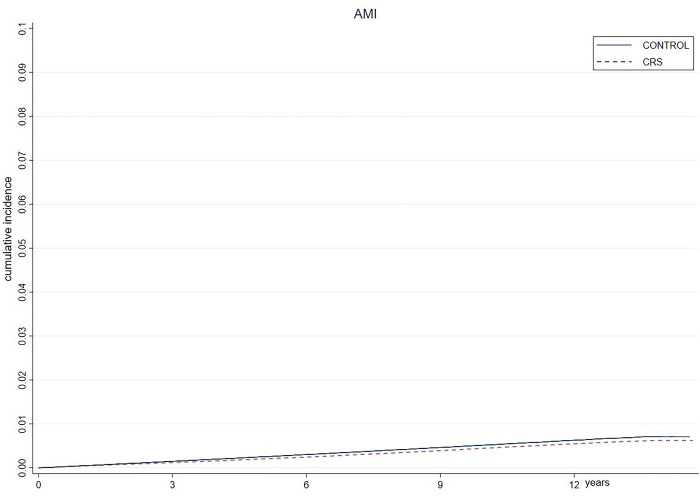
Kaplan-Meier plot of the incidence of acute myocardial infarction. Patients with CRS had a risk of developing AMI that was similar to that of the control group. AMI, acute myocardial infarction; CRS, chronic rhinosinusitis.

AMI did not occur more frequently in the CRS group than in the control group. The incidence rates of AMI were 8.23 and 8.28 per 1000 person-years in the CRS and control groups, respectively. The HR for AMI was not higher in the CRS group than in the control group (0.99 [0.97–1.02]). The HR for AMI did not differ according to sex compared with that in the control group (0.98 [0.95–1.01] and 1.01 [0.97–1.06] in males and females, respectively). Interestingly, the HR for AMI in the younger age group (aged < 50 years) was higher than that in the older age group (aged ≥ 50 years) (1.09 [1.03–1.15] in <50 years; 0.97 [0.94–0.99] in ≥50 years). The Kaplan–Meier plot revealed that the incidence of AMI was not higher in the CRS group than in the control group over time (Fig 2). The detailed results are presented in Table 3.

**Table 3:** Incidence rates of AMI events in the CRS and the control cohort according to the sex and age group.

Variables	Controls			Patients with CRS			HR (95% CI)
	Events	Person years	Incidence rate (95% CI)	Events	Person years	Incidence rate (95% CI)	
All	67917	81,998,848	8.28 (8.22–8.35)	6793	8,256,104	8.23 (8.03–8.43)	0.99 (0.97–1.02)
Sex							
Male	46898	41,977,014	11.17 (11.07–11.27)	4653	4238039.00	10.67 (10.67–11.30)	0.98 (0.95–1.01)
Female	21019	40,021,834	5.25 (5.18–5.32)	2140	4018065.00	5.33 (5.11–5.56)	1.01 (0.97–1.06)
Age (years)							
<50	13306	43607839	3.05 (3.00–3.10)	1455	4367993.4	3.33 (3.16–3.51)	1.09 (1.03–1.15)
≥50	54611	38391009	14.22 (14.11–14.34)	5338	3888110.7	13.73 (13.37–14.10)	0.97 (0.94–0.99)

The data are expressed as hazard ratio (95% confidence interval).

Abbreviations: AMI, acute myocardial infarction; CRS, chronic rhinosinusitis; CI, confidence interval; HR, hazard ratio.

### Incidence of AMI among patients with CRS according to NP

In the NP stratified analysis, there was no significant association between CRS and AMI, regardless of the presence of NP (adjusted HR [95% CI], 0.99 [0.97–1.02], 1.02 [0.98–1.05], and 0.97 [0.93–1.00] in the overall CRS, CRS without NP (CRSsNP), and CRS with NP (CRSwNP) groups, respectively). There were similar HRs according to sex and slightly increased HRs in the younger age group, regardless of the presence of NP. The Kaplan–Meier plot revealed that the incidence of AMI was not significantly different according to NP over time. The detailed results are presented in Table 4.

**Table 4:** Adjusted hazard ratios (95% CIs) of CRS for AMI according to the presence of NP.

Variables	Control	Overall CRS	CRSsNP	CRSwNP
Total	1 (Reference)	0.99 (0.97–1.02)	1.02 (0.98–1.05)	0.97 (0.93–1.00)
Sex				
Male	1 (Reference)	0.98 (0.95–1.01)	1.00(0.96–1.05)	0.96 (0.92–1.00)
Female	1 (Reference)	1.03 (0.98–1.08)	1.04 (0.99–1.10)	1.01 (0.93–1.08)
Age (years)				
<50	1 (Reference)	1.10 (1.04–1.16)	1.14 (1.05–1.23)	1.06 (0.99–1.151)
≥50	1 (Reference)	0.97 (0.94–1.00)	1.00 (0.96–1.04)	0.94 (0.90–0.98)

The data are expressed as hazard ratio (95% confidence interval). Adjusted variables: blood pressure, fasting blood sugar, cholesterol level, proteinuria, body mass index, alcohol consumption pattern, and smoking status.

Abbreviations: CRS, chronic rhinosinusitis; CRSsNP, chronic rhinosinusitis without nasal polyps; CRSwNP, chronic rhinosinusitis with nasal polyps.

## Discussion

In this nationwide cohort study, we identified 996,679 patients with incident CRS and 9,966,790 matched controls to evaluate the risk of AMI. This retrospective cohort study confirmed that there was no relationship between the two conditions. To the best of our knowledge, this study includes the largest, validated, and controlled cohort of patients with CRS with the goal of evaluating the risk of developing AMI. Notably, we found that patients with CRS were associated with better cardiovascular risk profiles than those in the control group participants. Therefore, the risk of AMI was not higher in the CRS group than in the control group. There was no sex-based association between CRS and AMI. In the younger age group, the risk of AMI was higher in the CRS group than in the controls. Considering that the CRS group had fewer cardiovascular risk factors than those of the controls, we believe that CRS was not an independent risk factor for AMI.

Several studies on the relationship between CRS and AMI have been conducted using population-based data. Previous studies reported a general increased risk of AMI in patients with CRS (adjusted HR [95% CI], 1.84 [1.44–2.40] [11], 1.48 [1.32–1.67] [12], and 1.29 [1.15–1.44] [13], respectively). On the other hand, the present study revealed no association between CRS and AMI (0.99 [0.97–1.02]). Previous studies were limited by insufficient control participants and vague definitions regarding risk factors [21, 22]. In contrast, we demonstrated that the risk of AMI in patients with CRS was not higher than that in controls when viewed after adjusting for cardiovascular risk profiles. Our analysis was performed based on incident cases using the NHSP data to reflect authentic clinical information. Recently, Jeon et al. [23] reported an increased risk of cardiovascular diseases in patients with CRS using the NHIS claim database. Their study was applied for relatively short period of wash-out (one year) to collect newly diagnosed patients with CRS. In addition, sample size of our study was larger than that of previous study (996,679 vs. 6,552, respectively). The different result would be made due to the methodological discordance.

Regarding cardiovascular risk profiles, no previous study has been well-adjusted for CRS. The present study showed that patients with CRS tend to have better risk factors, whereas previous studies indicated that CRS is associated with poor cardiovascular risk profiles [11, 12]. However, previous studies used claim codes as risk variables, which do not mirror factual clinical diagnoses. Our findings indicate that patients with CRS tend to demonstrate favorable cardiovascular risk profiles. These favorable risk factors imply that CRS itself might not overtly deteriorate the risk profiles of patients. Indeed, in the present study, patients with CRS showed healthier behaviors, including increased physical activity and lesser alcohol consumption as compared with those in matched controls. These healthy trends contrasted with previous findings showing that patients with CRS are more likely to be obese and prone to alcohol abuse [12]. In patients with CRS, the symptom complaints appear to be relatively mild. However, despite their mild symptoms, they received proportionally more medical services than the controls simply because they frequently visited the hospital due to CRS. Healthy behaviors may help deflect overt cardiovascular risk profiles, while the inherent pathophysiology of CRS can drive cardiovascular effects.

We analyzed the adjusted HR for AMI according to sex, age, hypertension, diabetes, dyslipidemia, proteinuria, drinking, and smoking status. Patients with hypertension, diabetes, and dyslipidemia are vulnerable to systemic diseases such as AMI. Alcohol consumption and smoking are well-known risk factors for AMI. Sex differences have been reported in patients with other upper airway diseases, such as obstructive sleep apnea, and this might be due to the existence of anatomical, hormonal, and endocrinological differences between sexes [24]. In our study, among patients with CRS, no significant difference was found for AMI according to sex.

Interestingly, younger patients with CRS showed an increased risk of AMI than that in older patients (1.09 [1.03–1.15] in <50 years). In terms of direct cardiovascular profiles, patients with CRS had a relatively lower proportion of risk factors (i.e., hypertension, high pulse rate, dyslipidemia, and high proteinuria) than that of controls. Hence, it is likely that the association between CRS and AMI is unrelated. Metabolic profiles, such as FBS and decreased physical activity, have also been considered essential risk factors for the development of cardiovascular diseases [25]. In our study, a slightly increased proportion of physical activity and a decreased proportion of FBS were observed in patients with CRS than those in controls. Likewise, the risk of AMI was not high in patients with CRS who had lower metabolic risk factors. It may be interpreted that patients with CRS frequently visited hospitals because of their chronic characteristics compared with that of the controls, which ultimately lead to lower cardiovascular and metabolic risk factors. Consequently, patients with CRS did not have an increased incidence of AMI compared with that of the control group. We found this outcome by controlling for important profiles in CRS for the risk of AMI, whereas previous studies did not adjust for these risk factors.

Statistically, we analyzed CRS and the comparison groups using standardized differences, and the HRs of AMI were calculated as the incidence cases per 1000 person-years. More importantly, a multivariable stratified Cox proportional hazard regression model was used for controlling the risk factors. Consequently, we were able to analyze the risk of AMI in patients with CRS in a large population (approximately 50 million people nationally).

The mechanism by which CRS contributes to AMI has been considered as multifactorial in the literature. First, CRS exerts a continuous, direct stimulus to the pharyngolaryngeal region due to postnasal drip (PND) [9, 26, 27]. Thus, the relationship between CRS and gastroesophageal reflux associated with PND has been reported [26–29]. Second, immunologically, CRS can continuously invoke chronic mucosal inflammation by releasing inflammatory cytokines into the peripheral airway mucosa. Goran et al. [30] noted that local inflammatory cytokines and chemokines might induce plaque growth, which may lead to local proteolysis, plaque rupture, and thrombus formation, which cause systemic ischemia and infarction. Third, physiologically, increased upper airway resistance can result in BP imbalance and increased cardiovascular risk. Similarly, an association of obstructive sleep apnea with AMI has been reported [31]. Therefore, CRS in the upper airway has been assumed to play a role in the development of AMI.

However, the above pathophysiological mechanisms are conjectured and lack literary evidence. We presume that the association between CRS and AMI is counterintuitive because of the anatomical distance. Pathologically, these two diseases have different mechanisms. AMI is characterized by myocardial necrosis due to ischemia in the coronary artery [32], whereas CRS is an inflammatory disease of the upper airway. Due to systematic discordance, the immunological impact of the upper airway system on the cardiovascular system has not yet been reported in the literature. More importantly, studies on cardiovascular risk profiles in patients with CRS are lacking. For these reasons, the relationship between CRS and AMI is inconclusive.

Meanwhile, CRS is not a local problem confined to the upper airway. Nasal polyposis is associated with lower airway disease; asthma, which in many cases is of greater severity; and an intolerance to nonsteroidal anti-inflammatory drugs [4, 33]. This fact can be understood as the unified airway concept [34], whereby related inflammatory cytokines act throughout the organism. Thus, shared inflammatory diseases of the upper and lower airways occur through overlapping disease processes [35].

NP is the primary phenotype that divides CRS into two subtypes (with and without NP) [3]. Clinically, CRSwNP is frequently associated with a negative impact on quality of life, unresponsiveness to medical therapy, and poorer prognosis after endoscopic sinus surgery compared with that of CRSsNP [36, 37]. Immunologically, CRSwNP tends to be skewed to the Th2 type inflammation [38, 39]. However, none of the previous studies regarding the relationship between CRS and AMI analyzed prognostic effect according to NP. In this study, we demonstrated that the presence of NP did not increase the risk of AMI in patients with CRS. Furthermore, sex did not affect the risk of AMI. However, among all CRS patients, the younger age group presented a slightly increased risk of AMI regardless of the presence of NP.

Based on our results, we are able to suggest several clinical benefits for decision making. First, physicians do not have to be under significant time pressure in deciding surgical management in patients with CRS with the goal of reducing the cardiovascular risk. Second, excessive concern about cardiac side effects from CRS drug interaction would be unnecessary during the decision making process for the medical management. Third, generally, anti-platelets such as Aspirin or Clopidogrel were recommended to be stopped in order to reduce the hemorrhage risk in the pre- and postoperative periods for surgery. Consequently, physicians do not have to shorten the stopping time of these drugs due to the uncertain additional cardiovascular risks. From clinical point of view, this study would be meaningful because an unnecessary concern regarding cardiovascular risk, such as AMI, could be eliminated when managing patients with CRS.

However, this study had several limitations. First, the CRS severity was not evaluated. This study was performed based on national claims data. Therefore, information regarding disease severity was lacking. Clinically, the severity of CRS has been determined by using CT (Lund-Mackay score) or endoscope (Lund-Kennedy score). Unfortunately, it is impossible to collect this information from the claim database. Therefore, we could not evaluate the effect of severity of CRS on the risk of AMI development in the present study. However, this is a common limitation in most previous studies on CRS, including randomized controlled studies. Second, we did not consider CRS recurrence even after surgical treatment. Cases of recurring CRS might provide different outcomes compared with those in primary cases. Finally, several variable, such as LDL cholesterol, was not provided by the database for the defined time in our study. Instead, we analyzed total cholesterol in lieu of LDL cholesterol as the risk factor. In the future, a large prospective trial is needed to overcome these limitations and verify the present outcomes.

## Conclusion

We found that the incidence of AMI was not increased in patients with CRS compared to that in controls by adjusting risk factor profiles using the NHIS claims database. In addition, the relationship between CRS and the incidence of AMI was denied regardless of the presence of NP. To the best of our knowledge, this is the first study controlling cardiovascular risk variables for analysis of the relationship between CRS and AMI. Clinically, these outcomes should be considered during diagnostic and therapeutic processes in patients with CRS for cardiovascular complications.

## Supporting information

S1 Table Diagnostic accuracy of chronic rhinosinusitis. (XLSX)
